# Supplementary material for: Multi-objective dung beetle optimization algorithm: A novel algorithm for solving complex multi-objective optimization problems
Source: PLoS One. 2025 Oct 14;20(10):e0331713. doi: 10.1371/journal.pone.0331713 (PMC12520404; doi:10.1371/journal.pone.0331713)
Supplement: Attachment — Submitted filename: Review.pdf [file pone.0331713.s005.pdf]

I appreciate the opportunity to review this manuscript, which presents the Multi-Objective Dung Beetle Optimization Algorithm (MODBO). The authors propose a novel approach to addressing complex multi-objective optimization problems (MOPs) by enhancing the original Dung Beetle Optimization (DBO) algorithm. Below are my comments and suggestions for improvement:

Novelty and Contribution: The integration of competitive and neighborhood mechanisms into MODBO is commendable. The comparative analysis against established algorithms adds considerable value to the study.

Methodological Rigor: The methodology is generally solid and well detailed.

Literature Positioning: The introduction provides a comprehensive overview of related works but lacks clarity in organization. Currently, it reads as a list rather than a cohesive narrative. A more structured presentation that clearly articulates how this study fits within the existing literature would strengthen the manuscript.

I must express my concern regarding the significant similarities between this manuscript and the foundational work titled "Dung beetle optimizer: a new meta-heuristic algorithm for global optimization." and derived works titled "Multi-Strategy Improved Dung Beetle Optimization Algorithm and Its Applications". Specific images (e.g., Image 1 and Image 3) and certain phrasings, such as the discussion surrounding the "No Free Lunch Theorem", page 3, and the description of the DBO algorithm, sec 2.1, reflect similar content and structure.

Given these overlaps, it is crucial that the authors clearly distinguish their contributions from this foundational work to avoid issues of dual publication or academic integrity.

Clarity and Writing Quality: Overall, the writing is clear, but results and conclusions sections could benefit from reorganization to enhance readability. Emphasizing key findings in the conclusion would provide a clearer takeaway for readers and highlight the study's significance.

Additionally, ensuring that all acronyms (e.g., MOEAs) are defined upon first use would improve accessibility for all readers. Typos are still present around the text.

Figures and Tables: While the figures and tables are informative, some appear raw. I recommend highlighting aggregated results and moving detailed data to an appendix to improve readability. It would also be beneficial to ensure that all figures have descriptive captions and that axes are appropriately labeled.

Limitations: Addressing potential limitations of the research in the conclusion would provide a more balanced view and further strengthen the manuscript.

Overall Recommendation In conclusion, I believe this manuscript makes a valuable and solid contribution to the field of optimization algorithms and has great potential for practical applications. I recommend acceptance with minor revisions, as I am confident that addressing the points mentioned will significantly enhance the quality and clarity of the work.

Thank you for considering my comments. I appreciate the effort the authors have put into this research and look forward to seeing how it develops.
