# Supplementary material for: Multi-objective dung beetle optimization algorithm: A novel algorithm for solving complex multi-objective optimization problems
Source: PLoS One. 2025 Oct 14;20(10):e0331713. doi: 10.1371/journal.pone.0331713 (PMC12520404; doi:10.1371/journal.pone.0331713)
Supplement: Attachment — Submitted filename: Response to Reviewers.docx [file pone.0331713.s003.docx]

**SUGGESTIONS FROM EDITOR**

**Academic Editor**

**Comment:** Thank you for submitting your manuscript to **PLOS ONE**. After careful consideration, we feel that it has merit but does not fully meet **PLOS ONE**’s publication criteria as it currently stands. Therefore, we invite you to submit a revised version of the manuscript that addresses the points raised during the review process.

**Response:** Thank you for your comments and suggestions concerning our manuscript. The comments and suggestions are all valuable and helpful for revising and improving our manuscript and the essential guiding significance to our research. We have studied the comments carefully and have made corrections.

**Comment #1:** Please ensure that your manuscript meets PLOS ONE's style requirements, including those for file naming. The PLOS ONE style templates can be found at

https://journals.plos.org/plosone/s/file?id=wjVg/PLOSOne_formatting_sample_main_body.pdf and

<https://journals.plos.org/plosone/s/file?id=ba62/PLOSOne_formatting_sample_title_authors_affiliations.pdf>

**Response #1:** Thank you for your comments. We have reformatted the paper to comply with the PLOS ONE requirements as per the document requirements.

**Comment #2:** Please note that PLOS ONE has specific guidelines on code sharing for submissions in which author-generated code underpins the findings in the manuscript. In these cases, all author-generated code must be made available without restrictions upon publication of the work. Please review our guidelines at https://journals.plos.org/plosone/s/materials-and-software-sharing#loc-sharing-code and ensure that your code is shared in a way that follows best practice and facilitates reproducibility and reuse.

Response #2: We uploaded the code and test functions involved in the paper to GitHub at: https://github.com/wu457120325/MIDBO_PLOSONE

**Comment #3:** We note that the grant information you provided in the ‘Funding Information’ and ‘Financial Disclosure’ sections do not match.

**Response #3:** Thank you for your comments. We have revalidated this section and made the changes.

**Additional Editor Comments**

**REVIEWER #1**

**Comment #1:** Does the proposed Multi-Objective Dung Beetle Optimization (MODBO) algorithm incorporate competitive and neighborhood mechanisms for solving multi-objective problems?

**Response #1:** Thank you for your comments on our article, and we would like to respond to your two questions as follows:

For the first question, we use the competition and neighborhood mechanisms in this article to optimize the MODBO algorithm. The purpose of using the competition mechanism is that the DBO algorithm relies heavily on the global and local optimums during the optimization process. Still, in a multi-objective optimization problem, the global optimums and local optimums are multiple vectors (Pareto front of the solutions of 1), and different solutions will have different effects on the particle guidance to ensure the diversity of the population; we choose the competition mechanism to guide the update of particle positions, which can be used to provide the diversity of the population. Different solutions will have different effects on particle guidance; to ensure the diversity of the population, we choose the competition mechanism to guide the update of particle positions, which can provide to the greatest extent that the particles will not converge prematurely and fall into the local optimum when they are approaching the best Pareto front. The neighborhood mechanism is proposed to improve the algorithm's robustness and ensure the uniformity of the solution because even if the search in some regions is not practical, the particles in the other areas can continue searching for possible solutions, increasing the possibility of finding the global optimal solution. We describe both mechanisms in detail in Sections 2.2.1 and 2.2.2.

Regarding your second question, there is a linkage between the two mechanisms, and Section 2.2.3 includes a linkage between the two mechanisms in the flow. During the optimization of the algorithm, the two mechanisms work together so that MODBO can show better performance in different multi-objective problems.

**Comment #2:** How does the introduction of fast, non-dominated sorting enhance the Dung Beetle Optimization Algorithm's ability to solve multi-objective optimization problems?

**Response #2:** Thank you for pointing this out and making us realize that we should have described this part of the paper. The original Dung Beetle Optimization algorithm is a single-objective algorithm; in the introduction, we explained some of the many applications of the DBO algorithm in engineering problems; based on its excellent single-objective capability, we reasonably deduced that it should also have a better optimization ability in multi-objective issues, so we improved it from a single-objective algorithm to a multi-objective algorithm. Still, from the single-objective algorithm to the multi-objective algorithm, the most critical problem to be solved is the particle ordering problem. In the original algorithm, due to the singularity of the objective, finding the optimal value only requires a simple comparison, but in the multi-objective algorithm, the optimal value is often a set of solutions in the form of curves or planes to find the closest solution set to the Pareto front of the objective function among the many solutions, it is necessary to introduce the nondominated sort, the nondominated sort can sort the vectors, giving different values to different vectors, so we improved it from the single-objective algorithm to the multi-objective algorithm. Vectors are sorted, giving different front values to other solutions, and based on the value of the front, it is possible to know which solution is better.

In the article, we add a description of non-dominated ordering in Section 2.2.1

**Comment #3:** What role does the Competition mechanism play in guiding global optimal search within the MODBO algorithm?

**Response #3:** The purpose of our proposed competitive mechanism is so that particles can find nearby optimal particles for their leadership as fast as possible. In traditional multi-objective optimization, the goal is to find a solution set that provides a good trade-off between different objectives. In contrast, the competition mechanism is proposed to maintain the diversity of the population and avoid premature convergence to a local optimum solution. By allowing competition between particles and other particles in the population, poorly performing particles are allowed to learn from better-performing particles, helping maintain the population's diversity and explore different regions of the solution space.

In the experimental section, we also describe the competition mechanism, which is explained from theoretical and experimental perspectives.

**Comment #4:** In the introduction, you need to connect the state of the art to your paper goals. Please follow the literature review by a clear and concise state of the art analysis. This should clearly show the knowledge gaps identified and link them to your paper goals. Please reason both the novelty and the relevance of your paper goals. Clearly discuss what the previous studies that you are referring to. What are the Research Gaps/Contributions? Please note that the paper may not be considered further without a clear research gap and novelty of the study.

Response #4: Thank you very much for your suggestion; we are aware of the lack of work described in the introduction and have added content to the introduction to briefly describe how the algorithms in this paper differ from other researched work and explain why this paper undertakes that work.

*Given that the DBO algorithm is effective, many practical improvements and applications have been proposed by many scholars. Zhu24 proposed three improvement strategies to enhance the diversity of the population and the slow convergence speed of the algorithm, using the Good Point Set strategy to increase the diversity and avoid falling into the local optimal solution, dynamically balancing the foraging dung beetles and the spawning dung beetles and improve the convergence factor: this can make the algorithm have better global search ability in the early stage and more vital local exploration ability in the late stage; improve the optimal spawning region in the original algorithm so that the algorithm can explore the solution space more fully*

*…*

**Comment #5:** Literature Review has the chance to be further improved: it seems that the authors have made the retrospection. However, via the review, what issues should be addressed? What is the current specific knowledge gap? What implication can be referred to? The above questions should be answered. Authors need to propose their study and compare it with A Multi Echelon Location-Routing-Inventory Model for a Supply Chain Network: NSGA II and Multi-Objective Whale Optimization Algorithm, A new modified social engineering optimizer algorithm for engineering applications.

**Response #5:** We agree with this comment. As you suggested, in the INSTRUCTION section, we also review some of the work of others and show the specific differences between our work and the work of others; in this paper, we address the multi-objective improvement of a potentially novel algorithm ----DBO algorithm, the algorithm has been available since 2023, most scholars have focused on the single-objective application of the algorithm and also with the direction of applications improvement in different directions, our proposed MODBO algorithm has good performance on a variety of test functions. It is validated on the sensor deployment problem. We also explain the differences between our paper and the one you recommended.

*Typically, the performance of a multi-objective algorithm depends on the performance of the original algorithm; the better the performance of the original algorithm, the better the potential for MOEA. Yannibelli17 combines multi-objective annealing algorithms with a multi-objective technique and demonstrates the algorithm's performance on a multi-objective scheduling problem. Multi-objective improvement of the Sine Cosine Algorithm to MOSCA by introducing non-dominated ordering and congestion distance in Tawhid18 and validating the rationality of the improvement in a disc brake design problem. The MOMPA, an improvement to the original Marine Predator Algorithm (MPA) in Zhong19, the method of elite selection is used to improve it, which was tested in CEC2019 and then also on numerous engineering problems; Liu20 optimized the multi-objective Gray Wolf optimizer(MOGWO) on the perforation parameters to reduce its sacrifice and increase the absorption ratio. Wang21 inherited the efficient evolutionary mechanism from Whale Optimization Algorithm (WOA) by combining Oppositional Learning (OBL) and Global Grid Ranking (GGR) mechanism into the multi-objective WOA, which was tested with benchmark functions and proved the effectiveness of its improvement. Mohammad Reza Pourhassan22 proposed a multi-objective-based integration model for solving the problem of location, routing, and inventory management of manufacturing centers, distribution centers, and transport vehicles considering the main objectives of cost minimization and maximization of drivers' working time and used two algorithms, MOWOA and NSGA-II, to pair solve this problem.*

**Comment #6:** How does the Neighborhood mechanism assist in guiding local optimal value search in the MODBO algorithm?

**Response #6:** Thank you for your question. The main reason for the proposal of the neighborhood mechanism is that the optimization process of the DBO algorithm relies too much on the guidance of the global optimum, especially in the reproduction process. In small dung beetles foraging in this case, the particles lose a certain degree of uniformity if they optimize towards a single reverse direction, and the introduction of the neighborhood mechanism, which allows each particle to refer to the information of particles within its neighborhood only, instead of the global optimal solution, can enhance the local search capability of the algorithm and limit the scope of information exchange between particles. It can improve the algorithm's local search ability, limit the scope of information exchange between particles, help discover more potential optimal solutions, and increase the chance for the algorithm to explore different solution space regions.

In the experimental part, we also describe the neighborhood mechanism, which explains the competition mechanism from theoretical and experimental perspectives.

**Comment #7:** What purpose does the external archive serve within the MODBO algorithm, and how does it contribute to achieving optimality?

**Response #7:** You have raised an important point. The Archive's role is to preserve all non-dominated solutions encountered, i.e., those solutions for which no other solution in the current solution set outperforms it in all objectives. These solutions form the set of approximate Pareto solutions. To keep the distribution of solutions as high as possible, the Archive needs to control the distribution of the stored solutions in the objective space, which is usually achieved through crowding calculations or other similarity measures to avoid overconcentration of solutions in a particular region. At the same time, due to the limited resources of the Archive, an upper limit of its stored solutions is controlled in the Archive to eliminate some of the solutions to maintain the size of the population. At the end of the algorithm, the set of solutions stored in Archive can be provided as the final output to the decision makers to help them weigh the different solutions and thus make a decision, which is the purpose of using Archive in our algorithm.

From single-objective to multi-objective algorithms, the use of Archive is one of the most fundamental parts used in most of the papers, so our description of Archive is put in the introduction.

**Comment #8:** How was the effectiveness of the MODBO algorithm evaluated, and what specific benchmarks were used in comparison with other algorithms?

**Response #8:** Thank you for your question. For the effectiveness of the MODBO algorithm, we chose to use two evaluation metrics, IGD and HV, to evaluate the effectiveness of our proposed algorithm. These two metrics are prevalent evaluation metrics of multi-objective algorithms for assessing the convergence distance and convergence accuracy of the multi-objective algorithms. On the CEC2020 test function, according to these two evaluation metrics, the performance of MODBO algorithm is due to the article of the ten algorithms compared in the article.

We add a discussion of the test functions in tabular form in Section 3.3.

Table 2. CEC2020

| **Function** | **Scalable number of variables** | **Scalable number of objectives** | **Pareto optima known** | **Pareto front geometry** | **Pareto set geometry** | **Scalable number of ops_local** | N_ops(N_global+N_local) |
| --- | --- | --- | --- | --- | --- | --- | --- |
| MMF1 | × | × | √ | Convex | Nonlinear | × | 2 + 0 |
| MMF2 | × | × | √ | Convex | Nonlinear | × | 2 + 0 |
| MMF4 | × | × | √ | Concave | Nonlinear | × | 2 + 0 |
| MMF5 | × | × | √ | Convex | Nonlinear | × | 2 + 0 |
| MMF7 | × | × | √ | Convex | Nonlinear | × | 2 + 0 |
| MMF8 | × | × | √ | Concave | Nonlinear | × | 2 + 0 |
| MMF10 | × | × | √ | Convex | Linear | × | 1 + 0 |
| MMF11 | × | × | √ | Convex | Linear | √ | 1 + 0 |
| MMF12 | × | × | √ | Convex | Linear | √ | 1 + 0 |
| MMF13 | × | × | √ | Convex | Nonlinear | √ | 1 + 0 |
| MMF14 | √ | √ | √ | Concave | Linear | √ | 2 + 0 |
| MMF15 | √ | √ | √ | Concave | Linear | √ | 1 + 0 |
| MMF1_e | × | × | √ | Convex | Nonlinear | × | 2 + 0 |
| MMF14_a | √ | √ | √ | Concave | Nonlinear | √ | 2 + 0 |
| MMF15_a | √ | √ | √ | Concave | Nonlinear | √ | 1 + 0 |
| MMF10_l | × | × | √ | Convex | Linear | × | 1 + 1 |
| MMF11_l | × | × | √ | Convex | Linear | √ | 1 + 1 |
| MMF12_l | × | × | √ | Convex | Linear | √ | 1 + 1 |
| MMF13_l | × | × | √ | Convex | Nonlinear | √ | 1 + 1 |
| MMF15_l | √ | √ | √ | Concave | Linear | √ | 1 + 1 |
| MMF15_a_l | √ | √ | √ | Concave | Nonlinear | √ | 1 + 1 |
| MMF16_l1 | √ | √ | √ | Concave | Linear | √ | 1 + 2 |
| MMF16_l2 | √ | √ | √ | Concave | Linear | √ | 2 + 1 |
| MMF16_l3 | √ | √ | √ | Concave | Linear | √ | 1 + 2 |

**Comment #9:** Can you explain how the MODBO algorithm performed when tested on engineering problems, such as the 3D sensor deployment problem?

**Response #9:** Thank you for your question. For the effectiveness of the MODBO algorithm, we chose to use two evaluation metrics, IGD and HV, to evaluate the effectiveness of our proposed algorithm. These two metrics are prevalent evaluation metrics of multi-objective algorithms for assessing the convergence distance and convergence accuracy of the multi-objective algorithms. On the CEC2020 test function, according to these two evaluation metrics, the performance of the MODBO algorithm is due to the article of the ten algorithms compared in the article.

We add a discussion of the test functions in tabular form in Section 3.3.

**Comment #10:** What are the key findings from the comparison of MODBO with other algorithms in terms of solving complex multi-objective problems, both in terms of convergence and distribution.

**Response #10:** Thank you for your question; our paper used three representative test functions to evaluate the distributivity and convergence of the MODBO algorithm in the solution space; Fig. 6-8 compares the convergence curves of 9 algorithms and MODBO on the test functions. The test functions are selected to consider both continuous and discontinuous problems, and it is demonstrated in Fig.6-8 that MODBO can find the solution better in the solution space and can utilize the competition mechanism to develop more PSs.The curves in Fig.9-11 represent that MODBO is more uniformly distributed on the solution space of the three different test functions, and the convergence curves of the final calculations are in good agreement with the standard convergence curves of the functions. The standard convergence curves of MODBO fit better.

**We greatly appreciate your excellent comments.**

**REVIEWER #2**

The article titled "Multi-objective dung beetle optimization algorithm: A novel algorithm for solving complex multi-objective optimization problems" introduces a new optimization algorithm, MODBO, which builds upon the existing dung beetle optimization algorithm (DBO) to address multi-objective problems (MOPs). This novel algorithm incorporates mechanisms such as competitive and neighborhood mechanisms, non-dominated sorting, and external archiving to enhance its capability to find optimal solutions in complex multi-objective spaces.

Key Components and Innovations:

1. Non-Dominated Sorting and External Archiving

Non-dominated sorting is a critical technique in multi-objective optimization that classifies solutions based on Pareto dominance. Solutions that are not dominated by any other solution form a Pareto front. MODBO integrates non-dominated sorting to efficiently manage and evolve the population of solutions. Additionally, an external archive is used to store the best non-dominated solutions found so far, ensuring that the algorithm maintains a diverse set of high-quality solutions across iterations.

2. Competition and Neighborhood Mechanisms

To enhance the search capabilities of the DBO algorithm, MODBO introduces two significant mechanisms:

• Competition Mechanism: This guides particles towards the global optimal solution by encouraging competition among particles. It helps in maintaining the diversity of the solutions and prevents premature convergence to suboptimal solutions.

• Neighborhood Mechanism: This mechanism focuses on local optimization by allowing particles to explore their immediate surroundings more thoroughly. It ensures that the algorithm can fine-tune solutions to achieve better local optimality.

3. Performance Evaluation and Benchmarking

The MODBO algorithm's performance is evaluated against nine established algorithms using the CEC2020 benchmark suite. Additionally, a practical application of MODBO is demonstrated through the 3D sensor deployment problem, which showcases its effectiveness in real-world scenarios.

Strengths:

1. Innovative Mechanisms for Improved Search

The incorporation of competition and neighborhood mechanisms addresses common issues in optimization algorithms, such as maintaining a balance between exploration and exploitation. These mechanisms ensure that the algorithm does not get trapped in local optima and can explore the solution space more effectively.

2. Comprehensive Benchmarking

The use of CEC2020 benchmark problems provides a robust and standardized means of evaluating the algorithm's performance. This comparison with established algorithms highlights the strengths and areas of improvement for MODBO.

3. Practical Application Demonstration

Applying MODBO to the 3D sensor deployment problem illustrates its practical utility and effectiveness in solving real-world problems. This practical validation adds credibility to the algorithm's theoretical advancements.

Weaknesses:

1. Complexity of Mechanisms

While the competition and neighborhood mechanisms enhance the algorithm's performance, they also add complexity. Implementing and tuning these mechanisms might require significant computational resources and expert knowledge, which could limit the algorithm's accessibility and usability.

2. Limited Scope of Applications Demonstrated

Although the 3D sensor deployment problem is a valid application, demonstrating MODBO's effectiveness across a wider range of practical problems would strengthen the claims about its versatility and robustness.

3. Dependence on Parameters

Like many optimization algorithms, MODBO's performance is highly dependent on the proper setting of various parameters (e.g., competition and neighborhood coefficients). Finding the optimal parameters can be challenging and may require extensive experimentation.

**Response:** Thank you for your professional comments on our article; we feel perfect. As far as you are concerned, several issues need to be addressed. Based on your valuable suggestions, we have made extensive changes to the previous draft based on your suggestions for improving the article, which are listed below.

**Comment #1:**  Simplification of Mechanisms

Exploring ways to simplify the competition and neighborhood mechanisms without significantly compromising performance could make the algorithm more accessible. For instance, adaptive mechanisms that automatically adjust parameters during the search process could reduce the need for manual tuning.

**Response #1:** Thank you very much for your suggestion. We have already considered this problem while writing the paper; for the competition mechanism and neighborhood mechanism added to the improved algorithm, we have tried our best to avoid hyper-parameters influence on the algorithm. For example, in the competition mechanism, the nearest optimal particles guide the particles. Still, in the multi-objective problem, there is more than one optimal particle, so we have to decide which particle to be guided by according to the angle between the optimal and guided particles. The angle between the optimal and guided particles decides which particle will be guided. In this process, all the operations are without hyperparameters. As for the neighborhood mechanism, the process is similar to a chain structure, combining each particle and two particles near it into a chain, in which there are also particles selecting their front and back particles on their own, without the need for manual parameterization.

**Comment #2:** Broader Application Testing

Extending the evaluation of MODBO to a wider range of real-world problems would provide more evidence of its generalizability and robustness. Including diverse applications from different domains such as logistics, finance, and bioinformatics would strengthen the case for MODBO's versatility.

**Response #2:** Thank you for this suggestion. It would have been interesting to explore this aspect. After the test functions in our paper and the application to sensors, we proved that our proposed algorithm has good performance. According to the "No free lunch" theorem, the algorithm performs well in sensors, but there is no guarantee that it performs similarly in other areas. In the future, we will explore the algorithms in different fields and propose improvement plans to enhance MODBO's versatility.

**Comment #3:** Parameter Sensitivity Analysis

Conducting a detailed sensitivity analysis of the algorithm's parameters would provide valuable insights into their impact on performance. This could help in developing guidelines or heuristics for setting parameters, making the algorithm more user-friendly.

**Response #3:** Thank you for your suggestion; as you said, the heuristic algorithm is very sensitive to the change of parameters. For different parameters, the optimization effect may be very different. According to your suggestion, we increased the optimization effect of different parameter settings of MODBO in the article to analyze the optimization effect and finally chose a better combination of parameters to test the function.

**Comment #4:** Integration with Machine Learning Techniques

Integrating MODBO with machine learning techniques could further enhance its performance. For example, using machine learning to predict the effectiveness of certain parameter settings or to guide the search process could improve both the efficiency and the outcomes of the optimization.

**Response #4:** Thank you for your suggestion; the development of heuristic algorithms has indeed been very much combined with machine learning techniques, and among these combined algorithms, heuristic algorithms to optimize the parameters of machine learning, such as alternative gradient descent methods, and there are also some articles that will let machine learning guide the search process of heuristic algorithms, and this part will be used as a part of our follow up study to continue exploring the relationship between MODBO and the various areas of computing combined.

**We greatly appreciate your excellent comments.**
