# Supplementary material for: Multi-objective dung beetle optimization algorithm: A novel algorithm for solving complex multi-objective optimization problems
Source: PLoS One. 2025 Oct 14;20(10):e0331713. doi: 10.1371/journal.pone.0331713 (PMC12520404; doi:10.1371/journal.pone.0331713)
Supplement: Attachment — Submitted filename: Response_to_Reviewers_auresp_2.docx [file pone.0331713.s004.docx]

**SUGGESTIONS FROM EDITOR**

**REVIEWER #2**

**Comment #1:**  The combined use of whale and moth-flame optimization algorithms.

This approach could potentially offer refined optimization techniques that could strengthen the analytical framework.

**Response #1:** Thank you very much for your suggestion. We noticed a flaw in our article: most of the algorithms compared in the article are multi-objective versions of the original algorithms, ignoring that many of the algorithms have been richly improved, and their performance increases when they become multi-objective algorithms. So, we added an experiment to the article to reproduce the algorithm proposed in the literature [32], in which we combined the WOA and MFO algorithms in a multi-objective variant, after which it was tested on 24 test functions. The results show a significant improvement compared to the original algorithm, but there is a gap compared to our proposed algorithm.

We modified both Table 2 and Table 3 and modified the experimental plots Fig.6-Fig.11

Table 3. IGD

| IGD |  | MO_Ring_PSO_SCD | NSGA2 | MOEA/D | SPEA2 | MOGWO | MOSCA | MOGTO | MOSSA | MOMMFO | MODBO |
| --- | --- | --- | --- | --- | --- | --- | --- | --- | --- | --- | --- |
| MMF1 | Mean | 1.97E-03 | 1.57E-03 | 4.12E-03 | 1.69E-03 | 1.18E-02 | 2.31E-03 | 3.02E-02 | 2.28E-03 | 1.77E-02 | 1.31E-03 |
|  | SD | 4.98E-05 | 1.30E-04 | 2.04E-04 | 9.15E-05 | 4.82E-03 | 6.61E-05 | 8.97E-03 | 8.30E-05 | 2.05E-03 | 6.95E-05 |
| MMF2 | Mean | 1.31E-02 | 8.12E-03 | 3.78E-03 | 1.26E-02 | 5.29E-02 | 1.45E-01 | 6.86E-02 | 5.58E-03 | 9.91E-02 | 1.53E-03 |
|  | SD | 1.74E-03 | 7.81E-03 | 4.58E-05 | 3.59E-03 | 2.37E-02 | 3.46E-02 | 2.04E-02 | 6.45E-04 | 2.41E-02 | 1.21E-04 |
| MMF4 | Mean | 1.76E-03 | 1.25E-03 | 3.81E-03 | 1.66E-03 | 1.68E-02 | 3.79E-03 | 2.83E-02 | 2.31E-03 | 1.24E-02 | 1.23E-03 |
|  | SD | 1.24E-04 | 6.03E-05 | 6.64E-06 | 1.35E-04 | 8.03E-03 | 1.90E-03 | 3.70E-03 | 8.73E-05 | 1.26E-03 | 7.93E-05 |
| MMF5 | Mean | 2.01E-03 | 1.32E-03 | 4.00E-03 | 1.70E-03 | 2.91E-03 | 2.25E-03 | 2.87E-02 | 2.22E-03 | 1.83E-02 | 1.28E-03 |
|  | SD | 5.14E-05 | 7.66E-05 | 1.13E-04 | 6.62E-05 | 3.10E-04 | 3.23E-05 | 7.62E-03 | 5.66E-05 | 1.50E-03 | 3.07E-05 |
| MMF7 | Mean | 1.88E-03 | 1.30E-03 | 3.84E-03 | 1.71E-03 | 5.39E-03 | 2.33E-03 | 2.47E-02 | 2.30E-03 | 1.30E-02 | 1.27E-03 |
|  | SD | 5.49E-05 | 6.57E-05 | 8.49E-06 | 1.22E-04 | 6.21E-04 | 4.00E-05 | 4.60E-03 | 5.36E-05 | 1.76E-03 | 4.27E-05 |
| MMF8 | Mean | 2.85E-03 | 1.26E-03 | 4.21E-03 | 1.65E-03 | 3.67E-03 | 2.55E-03 | 8.16E-02 | 2.33E-03 | 3.36E-02 | 1.30E-03 |
|  | SD | 1.95E-04 | 4.53E-05 | 3.37E-06 | 7.73E-05 | 3.93E-03 | 9.57E-05 | 1.84E-02 | 7.22E-05 | 5.62E-03 | 2.91E-05 |
| MMF10 | Mean | 1.07E-01 | 1.06E-01 | 2.02E-01 | 7.68E-02 | 5.07E-02 | 4.47E-02 | 3.19E-01 | 9.15E-02 | 2.46E-01 | 3.50E-03 |
|  | SD | 2.33E-02 | 1.01E-01 | 1.39E-01 | 7.22E-02 | 1.01E-02 | 6.42E-03 | 3.03E-02 | 2.59E-02 | 4.99E-02 | 1.34E-04 |
| MMF11 | Mean | 1.76E-02 | 1.09E-02 | 7.76E-02 | 1.38E-02 | 1.81E-02 | 9.13E-03 | 2.02E-01 | 9.41E-03 | 1.10E-01 | 5.01E-03 |
|  | SD | 1.31E-03 | 6.96E-04 | 1.23E-04 | 4.56E-04 | 1.96E-03 | 5.01E-04 | 1.83E-02 | 5.13E-04 | 1.34E-02 | 3.20E-04 |
| MMF12 | Mean | 7.65E-03 | 8.92E-03 | 6.72E-03 | 2.77E-03 | 5.09E-03 | 3.23E-02 | 9.82E-02 | 7.61E-03 | 7.31E-02 | 1.09E-03 |
|  | SD | 1.03E-03 | 2.12E-02 | 9.83E-06 | 1.19E-04 | 8.89E-04 | 3.95E-02 | 3.80E-02 | 1.88E-03 | 1.61E-02 | 4.55E-05 |
| MMF13 | Mean | 3.12E-02 | 1.37E-02 | 4.64E-01 | 1.68E-02 | 2.52E-02 | 1.18E-02 | 2.35E-01 | 1.64E-02 | 1.66E-01 | 6.41E-03 |
|  | SD | 2.34E-03 | 5.32E-04 | 5.59E-04 | 1.34E-03 | 3.54E-03 | 1.57E-04 | 5.26E-02 | 2.61E-03 | 1.99E-02 | 1.47E-04 |
| MMF14 | Mean | 6.90E-02 | 9.68E-02 | 2.35E-01 | 1.80E-01 | 1.41E-01 | 1.48E-01 | 2.60E-01 | 1.02E-01 | 1.42E-01 | 6.55E-02 |
|  | SD | 1.47E-03 | 3.64E-03 | 1.19E-02 | 2.00E-02 | 3.17E-02 | 5.65E-02 | 3.03E-02 | 3.05E-03 | 5.75E-03 | 2.30E-03 |
| MMF15 | Mean | 1.05E-01 | 1.45E-01 | 2.90E-01 | 2.39E-01 | 1.62E-01 | 1.04E-01 | 3.20E-01 | 1.09E-01 | 1.90E-01 | 6.63E-02 |
|  | SD | 5.05E-03 | 8.89E-03 | 2.00E-02 | 4.42E-02 | 4.94E-02 | 2.46E-02 | 2.51E-02 | 6.01E-03 | 1.45E-02 | 2.07E-03 |
| MMF1_e | Mean | 7.93E-03 | 8.06E-03 | 5.66E-02 | 1.02E-02 | 1.20E-02 | 3.42E-03 | 8.47E-02 | 3.02E-03 | 1.19E-01 | 1.63E-03 |
|  | SD | 1.00E-03 | 1.43E-02 | 1.69E-02 | 5.83E-03 | 4.20E-03 | 4.48E-04 | 3.76E-02 | 1.52E-04 | 2.51E-02 | 3.28E-04 |
| MMF14_a | Mean | 6.76E-02 | 1.00E-01 | 2.35E-01 | 2.07E-01 | 1.67E-01 | 1.85E-01 | 2.53E-01 | 1.04E-01 | 1.48E-01 | 6.65E-02 |
|  | SD | 1.37E-03 | 6.50E-03 | 1.94E-02 | 2.61E-02 | 2.27E-02 | 2.08E-02 | 2.43E-02 | 7.35E-03 | 8.08E-03 | 1.55E-03 |
| MMF15_a | Mean | 1.06E-01 | 1.65E-01 | 2.90E-01 | 2.96E-01 | 1.73E-01 | 2.05E-01 | 3.35E-01 | 1.10E-01 | 1.92E-01 | 6.75E-02 |
|  | SD | 4.38E-03 | 1.01E-02 | 2.05E-02 | 4.71E-02 | 3.49E-02 | 2.06E-02 | 3.21E-02 | 6.43E-03 | 9.64E-03 | 2.02E-03 |
| MMF10_I | Mean | 1.80E-01 | 1.97E-01 | 2.22E-01 | 2.01E-01 | 2.37E-01 | 2.16E-01 | 2.38E-01 | 2.31E-01 | 1.92E-01 | 1.92E-01 |
|  | SD | 1.40E-02 | 1.39E-02 | 1.34E-02 | 1.02E-02 | 1.90E-02 | 6.18E-03 | 2.88E-02 | 9.73E-03 | 3.92E-02 | 1.35E-04 |
| MMF11_I | Mean | 8.25E-02 | 9.14E-02 | 1.69E-01 | 9.41E-02 | 1.02E-01 | 9.31E-02 | 2.30E-01 | 9.30E-02 | 1.62E-01 | 9.07E-02 |
|  | SD | 6.38E-03 | 9.81E-05 | 8.08E-05 | 1.19E-03 | 1.81E-03 | 2.64E-04 | 3.01E-02 | 3.05E-04 | 2.04E-02 | 9.74E-05 |
| MMF12_I | Mean | 6.46E-02 | 8.22E-02 | 9.09E-02 | 7.99E-02 | 8.49E-02 | 1.06E-01 | 1.31E-01 | 8.88E-02 | 1.07E-01 | 8.22E-02 |
|  | SD | 1.28E-02 | 5.93E-05 | 6.63E-05 | 9.03E-03 | 1.44E-03 | 2.07E-02 | 1.12E-02 | 2.85E-03 | 1.43E-02 | 5.79E-05 |
| MMF13_I | Mean | 9.96E-02 | 1.45E-01 | 6.27E-01 | 1.49E-01 | 1.64E-01 | 1.47E-01 | 2.38E-01 | 1.45E-01 | 2.00E-01 | 1.44E-01 |
|  | SD | 2.61E-02 | 9.54E-04 | 3.78E-03 | 4.51E-03 | 8.18E-03 | 8.47E-04 | 5.22E-02 | 8.40E-03 | 4.63E-02 | 4.17E-04 |
| MMF15_I | Mean | 1.65E-01 | 2.03E-01 | 2.74E-01 | 2.61E-01 | 2.43E-01 | 2.30E-01 | 3.29E-01 | 1.90E-01 | 2.27E-01 | 1.78E-01 |
|  | SD | 3.28E-03 | 8.61E-03 | 1.35E-02 | 2.26E-02 | 3.11E-02 | 4.57E-02 | 2.51E-02 | 4.55E-03 | 8.69E-03 | 2.55E-03 |
| MMF15_a_I | Mean | 1.67E-01 | 1.97E-01 | 2.78E-01 | 2.86E-01 | 2.36E-01 | 2.51E-01 | 3.46E-01 | 1.96E-01 | 2.31E-01 | 1.79E-01 |
|  | SD | 4.08E-03 | 6.56E-03 | 9.45E-03 | 1.36E-02 | 3.92E-02 | 1.91E-02 | 2.39E-02 | 4.07E-03 | 8.62E-03 | 2.07E-03 |
| MMF16_l1 | Mean | 1.30E-01 | 1.64E-01 | 2.82E-01 | 2.45E-01 | 2.08E-01 | 2.25E-01 | 3.35E-01 | 1.65E-01 | 2.22E-01 | 1.47E-01 |
|  | SD | 3.22E-03 | 5.93E-03 | 1.90E-02 | 2.77E-02 | 3.57E-02 | 7.59E-02 | 3.82E-02 | 4.45E-03 | 8.63E-03 | 1.75E-03 |
| MMF16_l2 | Mean | 2.02E-01 | 2.43E-01 | 2.74E-01 | 2.89E-01 | 2.78E-01 | 2.52E-01 | 3.57E-01 | 2.27E-01 | 2.50E-01 | 2.31E-01 |
|  | SD | 4.92E-03 | 7.51E-03 | 1.41E-02 | 1.28E-02 | 3.05E-02 | 4.02E-02 | 3.11E-02 | 3.82E-03 | 8.37E-03 | 1.69E-03 |
| MMF16_L3 | Mean | 1.63E-01 | 1.98E-01 | 2.78E-01 | 2.65E-01 | 2.32E-01 | 2.52E-01 | 3.46E-01 | 1.96E-01 | 2.37E-01 | 1.89E-01 |
|  | SD | 4.26E-03 | 5.08E-03 | 1.61E-02 | 2.31E-02 | 3.17E-02 | 4.82E-02 | 2.96E-02 | 5.20E-03 | 1.44E-02 | 2.77E-03 |

Table 4. HV value

| HV |  | MO_Ring_PSO_SCD | NSGA2 | MOEA/D | SPEA2 | MOGWO | MOSCA | MOGTO | MOSSA | MOMMFO | MODBO |
| --- | --- | --- | --- | --- | --- | --- | --- | --- | --- | --- | --- |
| MMF1 | Mean | 8.73E-01 | 8.74E-01 | 8.65E-01 | 8.74E-01 | 8.56E-01 | 8.73E-01 | 8.07E-01 | 8.73E-01 | 8.44E-01 | 8.75E-01 |
|  | SD | 1.32E-04 | 4.77E-04 | 7.84E-03 | 2.84E-04 | 8.16E-03 | 1.38E-04 | 2.76E-02 | 7.85E-05 | 4.38E-03 | 3.17E-05 |
| MMF2 | Mean | 8.59E-01 | 8.66E-01 | 8.71E-01 | 8.54E-01 | 8.13E-01 | 6.32E-01 | 5.64E-01 | 8.68E-01 | 6.37E-01 | 8.74E-01 |
|  | SD | 2.28E-03 | 7.14E-03 | 5.96E-05 | 8.00E-03 | 2.15E-02 | 6.75E-02 | 2.18E-01 | 1.04E-03 | 6.19E-02 | 2.00E-04 |
| MMF4 | Mean | 5.40E-01 | 5.42E-01 | 5.36E-01 | 5.40E-01 | 5.20E-01 | 5.38E-01 | 4.73E-01 | 5.40E-01 | 5.15E-01 | 5.42E-01 |
|  | SD | 3.59E-04 | 6.24E-05 | 5.63E-03 | 4.32E-04 | 1.16E-02 | 3.01E-03 | 2.56E-02 | 3.70E-05 | 3.43E-03 | 6.29E-05 |
| MMF5 | Mean | 8.73E-01 | 8.75E-01 | 8.68E-01 | 8.74E-01 | 8.72E-01 | 8.73E-01 | 8.21E-01 | 8.73E-01 | 8.42E-01 | 8.75E-01 |
|  | SD | 1.19E-04 | 7.08E-05 | 4.87E-03 | 2.77E-04 | 3.93E-04 | 1.29E-04 | 2.40E-02 | 1.00E-04 | 5.82E-03 | 5.38E-05 |
| MMF7 | Mean | 8.74E-01 | 8.75E-01 | 8.71E-01 | 8.74E-01 | 8.69E-01 | 8.73E-01 | 8.36E-01 | 8.73E-01 | 8.54E-01 | 8.75E-01 |
|  | SD | 1.44E-04 | 1.73E-04 | 4.70E-05 | 2.06E-04 | 9.45E-04 | 7.06E-05 | 4.59E-03 | 1.02E-04 | 2.79E-03 | 4.42E-05 |
| MMF8 | Mean | 4.19E-01 | 4.23E-01 | 4.20E-01 | 4.20E-01 | 4.19E-01 | 4.21E-01 | 2.98E-02 | 4.22E-01 | 3.03E-01 | 4.23E-01 |
|  | SD | 9.83E-04 | 2.70E-05 | 2.51E-05 | 1.04E-03 | 5.98E-03 | 3.99E-04 | 2.77E-01 | 1.34E-04 | 6.03E-02 | 1.05E-04 |
| MMF10 | Mean | 1.26E+01 | 1.23E+01 | 1.20E+01 | 1.27E+01 | 1.27E+01 | 1.28E+01 | 1.13E+01 | 1.27E+01 | 1.17E+01 | 1.29E+01 |
|  | SD | 8.27E-02 | 3.78E-01 | 7.20E-01 | 2.70E-01 | 3.45E-02 | 2.00E-02 | 8.35E-02 | 5.31E-02 | 1.22E-01 | 1.85E-04 |
| MMF11 | Mean | 1.45E+01 | 1.45E+01 | 1.44E+01 | 1.45E+01 | 1.45E+01 | 1.45E+01 | 1.34E+01 | 1.45E+01 | 1.41E+01 | 1.45E+01 |
|  | SD | 4.71E-03 | 1.29E-03 | 3.37E-04 | 3.67E-03 | 7.65E-03 | 5.08E-04 | 1.85E-01 | 8.70E-04 | 8.66E-02 | 1.96E-04 |
| MMF12 | Mean | 1.56E+00 | 1.57E+00 | 1.57E+00 | 1.57E+00 | 1.57E+00 | 1.47E+00 | 1.45E+00 | 1.55E+00 | 1.35E+00 | 1.57E+00 |
|  | SD | 9.55E-03 | 1.40E-01 | 1.28E-05 | 6.38E-04 | 8.54E-04 | 1.62E-01 | 5.35E-02 | 1.58E-02 | 1.38E-01 | 1.32E-05 |
| MMF13 | Mean | 1.84E+01 | 1.84E+01 | 1.70E+01 | 1.84E+01 | 1.84E+01 | 1.84E+01 | 1.72E+01 | 1.84E+01 | 1.76E+01 | 1.85E+01 |
|  | SD | 1.32E-02 | 9.99E-04 | 3.06E-03 | 5.72E-03 | 1.00E-02 | 7.54E-04 | 1.31E-01 | 1.73E-02 | 1.08E-01 | 3.67E-04 |
| MMF14 | Mean | 2.98E+00 | 2.83E+00 | 2.38E+00 | 2.60E+00 | 3.56E+00 | 3.11E+00 | 2.87E+00 | 2.82E+00 | 2.72E+00 | 2.83E+00 |
|  | SD | 2.20E-01 | 6.01E-02 | 3.35E-01 | 4.46E-01 | 6.45E-01 | 9.46E-01 | 3.24E-01 | 7.03E-02 | 3.16E-01 | 4.38E-02 |
| MMF15 | Mean | 4.01E+00 | 4.11E+00 | 3.45E+00 | 3.82E+00 | 5.10E+00 | 4.17E+00 | 3.93E+00 | 4.18E+00 | 3.89E+00 | 4.19E+00 |
|  | SD | 1.25E-01 | 5.94E-02 | 2.28E-01 | 3.97E-01 | 6.18E-01 | 2.56E-01 | 3.97E-01 | 7.02E-02 | 2.28E-01 | 6.00E-02 |
| MMF1_e | Mean | 8.53E-01 | 8.67E-01 | -7.49E+01 | 8.52E-01 | 8.56E-01 | 8.68E-01 | -1.20E+00 | 8.72E-01 | -7.30E-01 | 8.74E-01 |
|  | SD | 8.84E-03 | 1.11E-02 | 2.27E+01 | 1.91E-02 | 6.90E-03 | 5.81E-03 | 2.25E+00 | 4.49E-04 | 1.04E+00 | 6.95E-04 |
| MMF14_a | Mean | 2.89E+00 | 2.86E+00 | 2.47E+00 | 2.60E+00 | 3.36E+00 | 3.15E+00 | 2.79E+00 | 2.86E+00 | 2.83E+00 | 2.88E+00 |
|  | SD | 1.84E-01 | 5.47E-02 | 5.01E-01 | 4.52E-01 | 5.81E-01 | 2.54E-01 | 3.04E-01 | 6.45E-02 | 4.09E-01 | 3.97E-02 |
| MMF15_a | Mean | 4.08E+00 | 4.61E+00 | 3.56E+00 | 3.59E+00 | 4.18E+00 | 4.55E+00 | 3.57E+00 | 4.30E+00 | 3.92E+00 | 4.41E+00 |
|  | SD | 8.74E-02 | 1.43E-01 | 3.24E-01 | 4.31E-01 | 7.15E-01 | 6.28E-01 | 2.64E-01 | 5.93E-02 | 3.25E-01 | 4.21E-02 |
| MMF10_I | Mean | 1.27E+01 | 1.27E+01 | 1.22E+01 | 1.29E+01 | 1.27E+01 | 1.28E+01 | 1.13E+01 | 1.27E+01 | 1.16E+01 | 1.29E+01 |
|  | SD | 2.19E-02 | 1.99E-01 | 7.17E-01 | 2.87E-02 | 3.98E-02 | 2.07E-02 | 8.08E-02 | 4.44E-02 | 1.05E-01 | 1.26E-04 |
| MMF11_I | Mean | 1.45E+01 | 1.45E+01 | 1.44E+01 | 1.45E+01 | 1.45E+01 | 1.45E+01 | 1.36E+01 | 1.45E+01 | 1.41E+01 | 1.45E+01 |
|  | SD | 1.55E-03 | 4.11E-04 | 2.18E-04 | 2.68E-03 | 4.43E-03 | 4.98E-04 | 1.27E-01 | 5.94E-04 | 7.44E-02 | 3.90E-04 |
| MMF12_I | Mean | 1.57E+00 | 1.57E+00 | 1.57E+00 | 1.57E+00 | 1.57E+00 | 1.48E+00 | 1.45E+00 | 1.56E+00 | 1.41E+00 | 1.57E+00 |
|  | SD | 3.79E-03 | 4.67E-05 | 1.07E-05 | 5.18E-04 | 6.14E-04 | 1.30E-01 | 5.57E-02 | 6.51E-03 | 7.26E-02 | 1.97E-05 |
| MMF13_I | Mean | 1.84E+01 | 1.85E+01 | 1.68E+01 | 1.84E+01 | 1.84E+01 | 1.84E+01 | 1.71E+01 | 1.84E+01 | 1.77E+01 | 1.85E+01 |
|  | SD | 7.66E-03 | 2.39E-04 | 5.95E-01 | 2.33E-03 | 1.13E-02 | 7.11E-04 | 1.24E-01 | 2.75E-02 | 8.62E-02 | 1.87E-04 |
| MMF15_I | Mean | 4.93E+00 | 4.21E+00 | 3.58E+00 | 3.99E+00 | 4.27E+00 | 4.52E+00 | 4.08E+00 | 4.24E+00 | 3.87E+00 | 4.21E+00 |
|  | SD | 1.82E-01 | 8.79E-02 | 3.79E-01 | 2.81E-01 | 5.27E-01 | 9.64E-01 | 4.17E-01 | 1.05E-01 | 4.54E-01 | 5.42E-02 |
| MMF15_a_I | Mean | 4.26E+00 | 4.26E+00 | 3.51E+00 | 3.73E+00 | 4.27E+00 | 4.43E+00 | 3.54E+00 | 4.26E+00 | 3.76E+00 | 4.57E+00 |
|  | SD | 8.45E-02 | 7.32E-02 | 3.59E-01 | 3.41E-01 | 5.54E-01 | 4.05E-01 | 3.73E-01 | 7.63E-02 | 2.68E-01 | 5.45E-02 |
| MMF16_l1 | Mean | 4.28E+00 | 4.24E+00 | 3.74E+00 | 3.89E+00 | 4.20E+00 | 3.80E+00 | 4.23E+00 | 4.55E+00 | 3.79E+00 | 4.27E+00 |
|  | SD | 3.56E-01 | 4.53E-02 | 3.00E-01 | 4.09E-01 | 6.83E-01 | 5.69E-01 | 3.57E-01 | 1.05E-01 | 3.25E-01 | 6.61E-02 |
| MMF16_l2 | Mean | 4.15E+00 | 4.26E+00 | 3.58E+00 | 4.00E+00 | 4.74E+00 | 4.31E+00 | 3.96E+00 | 4.23E+00 | 3.96E+00 | 4.23E+00 |
|  | SD | 2.29E-01 | 5.18E-02 | 2.12E-01 | 2.49E-01 | 4.46E-01 | 6.36E-01 | 6.16E-01 | 1.05E-01 | 2.42E-01 | 4.43E-02 |
| MMF16_l3 | Mean | 4.43E+00 | 4.24E+00 | 3.54E+00 | 3.82E+00 | 4.23E+00 | 4.48E+00 | 3.89E+00 | 4.21E+00 | 3.87E+00 | 4.80E+00 |
|  | SD | 1.19E-01 | 2.63E-02 | 4.82E-01 | 2.84E-01 | 4.50E-01 | 1.05E+00 | 4.21E-01 | 9.34E-02 | 3.54E-01 | 4.31E-02 |

*[32]Nadimi-Shahraki MH, Fatahi A, Zamani H, et al. Hybridizing of Whale and Moth-Flame Optimization Algorithms to Solve Diverse Scales of Optimal Power Flow Problem. Electronics 2022;11(5):831; doi: 10.3390/electronics11050831.*

**Comment #2:** An optimal task scheduling method in Fog-IoT networks, utilizing a combination of AO and Whale Optimization Algorithm (WOA).

This combination may provide valuable insights into improving the efficiency and effectiveness of task management within the Fog-IoT environment.

**Response #2:** Thank you for your valuable comments and interest in our paper. You mentioned the idea of “AO and Whale Optimization Algorithm (WOA)-based Optimization Approach for Task Scheduling in Fog-IoT Networks.” We have less elaboration on this in Section IV, so we have added a description of this section at the beginning of Section IV, with corresponding references, and likewise, our contribution to sensor research. Our research focuses on the sensor deployment problem rather than task scheduling in Fog-IoT. Our work focuses on how to study sensor deployment in complex 3D environments using the MODBO algorithm for more efficient data collection and monitoring. We believe this approach can better utilize the performance of sensors and applies to real-world scenarios.

*In this section, the MODBO algorithm will be used to verify its effectiveness in complex engineering problems, and we apply the proposed algorithm to the sensor deployment problem. Swarm intelligence algorithms have had numerous applications in sensor problems, and Konstantinidis38 proposed a decomposition-based multi-objective evolutionary algorithm (MOEA/D) and a problem-specific generalized subproblem-related heuristic (GSH) algorithm and defined the dense deployment and power allocation problem (d-DPAP) in wireless sensor networks (WSNs). The d-DPAP is decomposed into scalar subproblems with parallel optimization subproblems. xiong39 considered the network lifetime and coverage of WSNs to repair the coverage holes using a multi-objective particle swarm optimization algorithm with an adaptive mesh and used an improved binary multi-objective evolutionary algorithm based on undominated sorting and bi-directional local search to schedule sensor nodes into non-disjoint subsets to extend the network lifetime. Shally Gupta40 proposed an enhanced deep reinforcement learning algorithm based on dynamic load balancing to improve load balancing performance in fog computing in IoT environments. The algorithm combines Grey Wolf optimization（GWO） and modified Moth Flame algorithm（MFO） and performs well in terms of throughput, latency, response time, and energy consumption. It is also more efficient than existing techniques. In contrast, this paper proposes a 3D sensor deployment method that can be deployed in blind areas in 3D scenes and validated in complex scenes. To confirm the high adaptability of the MODBO algorithm compared to other algorithms, we compare the MODBO algorithm with MOEAD, MOSCA and MOSSA algorithms using the same parameters as in the previous section.*

**We greatly appreciate your excellent comments.**
