# Supplementary material for: Multi-objective dung beetle optimization algorithm: A novel algorithm for solving complex multi-objective optimization problems
Source: PLoS One. 2025 Oct 14;20(10):e0331713. doi: 10.1371/journal.pone.0331713 (PMC12520404; doi:10.1371/journal.pone.0331713)
Supplement: Attachment — Submitted filename: Response_to_Reviewers_auresp_3.docx [file pone.0331713.s006.docx]

**SUGGESTIONS FROM EDITOR**

**Comment #1:**  Page 3: The introduction of the term "multi-objective" could be clarified earlier in the text to enhance reader understanding.

**Response #1:** Thanks to your comments, we have included an explanation of multi-targeting at the top of the abstract so that the reader understands the work of our article from the very beginning of reading it.

**Comment #2:**  Page 4: The phrase "leveraging the effectiveness of the dung beetle optimization algorithm" should be rephrased for clarity and conciseness.

**Response #2:** Thank you for your comment. We have reworded the sentence, and the revised sentence reads, " Thanks to the dung beetle optimization algorithm's fast convergence and robust optimization finding ability in single-objective optimization algorithms."

**Comment #3:**  Page 5: Consider providing a brief explanation of the "Competition mechanism" and "Neighborhood mechanism" to ensure all readers grasp their significance.

**Response #3:** Thanks to your comment, we have added an explanation of the two mechanisms at the beginning of section 2.

*In this section, we explain the DBO algorithm and optimize the MODBO algorithm using the competition and neighborhood mechanisms. The purpose of using the competition mechanism is that the DBO algorithm relies heavily on the global and local optimums during the optimization process. Still, in a multi-objective optimization problem, the global optimums and local optimums are multiple vectors (Pareto front of the solutions of 1), and different solutions will have different effects on the particle guidance to ensure the diversity of the population; we choose the competition mechanism to guide the update of particle positions, which can be used to provide the diversity of the population. Different solutions will have different effects on particle guidance; to ensure the diversity of the population, we choose the competition mechanism to guide the update of particle positions, which can provide to the greatest extent that the particles will not converge prematurely and fall into the local optimum when they are approaching the best Pareto front. The neighborhood mechanism is proposed to improve the algorithm's robustness and ensure the uniformity of the solution because even if the search in some regions is not practical, the particles in the other areas can continue searching for possible solutions, increasing the possibility of finding the global optimal solution. We describe both mechanisms in detail in Sections 2.2.1 and 2.2.2.*

**Comment #4:**  Page 6: There is a typographical error in the sentence discussing "non-dominated sorting"; please correct it to maintain professionalism.

**Response #4:** Thanks to your comment, we have corrected “non-dominated sorting” throughout the article to ensure uniformity of expression.

**Comment #5:**  Page 7: The reference to "CEC2020" should include a brief description of what it entails for readers unfamiliar with it.

**Response #5:** Thanks to your comments, we have redone the explanation of CEC2020 at the beginning of section 3.

*In this section, we perform several experiments to investigate the performance of MODBO in solving large-scale MOPs. Functional tests have been conducted in this section using 24 test functions from CEC2020, a collection of test functions specifically designed for evaluating multi-objective optimization algorithms. These test functions simulate various properties in real-world problems, such as nonlinearity, modality, rotation, translation, and noise, to comprehensively evaluate the capabilities of the algorithms. The CEC2020 test function set contains a series of problems with different dimensions, complexities, and properties to test the algorithms' convergence, diversity preservation, and robustness. These include 14 bi-objective test functions and ten tri-objective test functions. The MODBO algorithm is compared with one of the nine algorithms in Table 1, using PS and PF plots to visualize the algorithm's performance.*

**Comment #6:**  Page 8: The transition between sections could be smoother; consider adding a sentence to link the discussion of algorithms to the results presented.

**Response #5:** Thank you for your comment. We have reworked the connections between sentences to make the article smoother.

**Comment #7:** Page 9: The table formatting in Table 2 is inconsistent; ensure all columns are aligned for better readability.

**Response #7:** Thanks for your comment; we have reformatted Table 2 to ensure a better reading experience.

**Comment #8:**  Page 10: The experimental results could benefit from a more detailed explanation of the metrics used for evaluation.

**Response #8:** Thanks to your comments, we have explained the evaluated metrics in more detail to show the experimental results.

**Comment #9:**  Page 11: The conclusion could be strengthened by summarizing the implications of the findings more explicitly.

**Response #9:**  Thanks to your comments, We have added the meaning of indicators to the discussion of the paper's experimental results, which can strengthen the conclusions more clearly.

**Comment #10:**  Page 12: Ensure all figures are referenced in the text before they appear to guide the reader effectively through the manuscript.

**Response #10:** Thanks to your comments, We have validated all figures in the full text to ensure that all figures are cited in the text.

**We greatly appreciate your excellent comments.**
