# Supplementary material for: Multi-objective dung beetle optimization algorithm: A novel algorithm for solving complex multi-objective optimization problems
Source: PLoS One. 2025 Oct 14;20(10):e0331713. doi: 10.1371/journal.pone.0331713 (PMC12520404; doi:10.1371/journal.pone.0331713)
Supplement: Attachment — Submitted filename: Response_to_Reviewers_auresp_4.docx [file pone.0331713.s007.docx]

**SUGGESTIONS FROM EDITOR**

**Editor:** Please review your reference list to ensure that it is complete and correct. If you have cited papers that have been retracted, please include the rationale for doing so in the manuscript text or remove these references and replace them with relevant current references. Any changes to the reference list should be mentioned in the rebuttal letter that accompanies your revised manuscript. If you need to cite a retracted article, indicate the article’s retracted status in the References list and also include a citation and full reference for the retraction notice.

**Response #1:** We have checked all the references and ensured that the format of the references meets the requirements of PLOS ONE, and we have replaced any problematic references.

**REVIEWER #3**

**Comment #1:** The introduction provides a comprehensive overview of related works but lacks clarity in organization. Currently, it reads as a list rather than a cohesive narrative. A more structured presentation that clearly articulates how this study fits within the existing literature would strengthen the manuscript.

**Response #1:** Thank you for your comment. We have added a guiding narrative in the introduction to introduce our work and clarify the differences between existing work and our paper.

**Comment #2:** I must express my concern regarding the significant similarities between this manuscript and the foundational work titled "Dung beetle optimizer: a new meta-heuristic algorithm for global optimization." and derived works titled "Multi-Strategy Improved Dung Beetle Optimization Algorithm and Its Applications". Specific images (e.g., Image 1 and Image 3) and certain phrasings, such as the discussion surrounding the "No Free Lunch Theorem", page 3, and the description of the DBO algorithm, sec 2.1, reflect similar content and structure.Given these overlaps, it is crucial that the authors clearly distinguish their contributions from this foundational work to avoid issues of dual publication or academic integrity.

**Response #2:** Thank you for your comments. We have carefully read the two references you provided and can point out significant differences between our article and these two articles. 1. Our article focuses on solving multi-objective problems, and the strategies proposed serve multi-objective functions, while these two articles focus more on single-objective algorithms. If we limit the goal of solving problems in our article to a single objective, our proposed algorithm can solve single-objective problems. However, the algorithms proposed in these two articles cannot solve multi-objective problems 2. The engineering problems solved in our article mainly focus on deploying sensors in 3D environments and explain the problems faced in deployment, which are closely related to reality. The problems tested in these two articles are more common standard test datasets such as Welded Beam Design Issues and Reducer Design Issues 3. As we are all making changes to the original DBO algorithm, there may be some similarities in the description of the original algorithm. However, our improvements on the original algorithm are different. The improvement strategy proposed in our article focuses more on the algorithm handling complex multi-objective problems, while these two articles focus on handling single-objective problems.

**Comment #3:** Overall, the writing is clear, but results and conclusions sections could benefit from reorganization to enhance readability. Emphasizing key findings in the conclusion would provide a clearer takeaway for readers and highlight the study’s significance. Additionally, ensuring that all acronyms (e.g., MOEAs) are defined upon first use would improve accessibility for all readers. Typos are still present around the text.

**Response #3:** Thank you for your comments. We have revised the wording of the conclusion section to make it more straightforward. We have checked all abbreviations in the article and provided explanations for them.

**Comment #4:** While the figures and tables are informative, some appear raw. I recommend highlighting aggregated results and moving detailed data to an appendix to improve readability. It would also be beneficial to ensure that all figures have descriptive captions and that axes are appropriately labeled.

**Response #4:** Thank you for your comments. We have rewritten the titles of some charts to ensure that they are descriptive and explained in the article.

**Comment #5:** Addressing potential limitations of the research in the conclusion would provide a more balanced view and further strengthen the manuscript.

**Response #5:** Thank you for your comments. We added Limitations in Section 4.5, which elaborated on the remaining issues with our algorithm in the article and provided further plans for future research.

**Reviewer #4:**

**Comment #1:** There exists already MODBO using non dominated sorting algorithm in the literature. Go through the following link https://doi.org/10.1016/j.heliyon.2024.e37286. How you method is different and what is need of proposing the same method.

**Response #1:** Thank you for your comment on our article. There are some similarities in the methods used in both articles. We introduced a non-dominated sorting mechanism to improve the beetle optimization algorithm to multi-objective. However, the article （https://doi.org/10.1016/j.heliyon.2024.e37286) is a sorting method used by the ancient algorithm NSGA-II. However, this article uses a newly proposed non-dominated sorting method in recent years, which reduces time complexity and has a better sorting ability than that article.

Regarding algorithm improvement, our paper idea and article aim to solve the problem of the beetle algorithm quickly getting stuck in local optima. Four improvement strategies were used in that article using the CF series test functions. Our article used two improvement strategies, using MMF series test functions. CF series test functions mainly focus on multi-objective optimization problems with constraints, while MMF test functions focus on multi-objective optimization problems without constraints but with multimodal characteristics. CF test functions are closer to practical engineering applications, while MMF test functions are more commonly used in academic research to examine the performance of algorithms in complex search spaces. Our goals are mainly set for engineering problems. The article examines the flight capability of drones under various constrained conditions. In contrast, our article aims to improve the population's distribution ability to ensure the integrity of sensor coverage.

**Comment #2:** Instead of using CEC 2020 test suit can the authors use the recent test suits for instance CEC 2022.

**Response #2:** Thank you for your comment on our article. CEC2020 includes 24 test functions, including tests in various multi-objective scenarios, including functions with complex multimodal frontiers, multiple local optima, and discontinuous Pareto frontiers, which can comprehensively test the ability of our algorithm. CEC2022 is mainly used to test the performance of single-objective algorithms. At the same time, CEC2023 focuses more on testing dynamic multi-objective algorithms, which is also one of the tasks we will be doing soon.

**Comment #3:** The comparative study is messing in the manuscript with respect to the technique. It is advised to test and compare with the above reference if both are different.

**Response #3:** Thank you for your comments. In comparing algorithms, we selected MMF1, MMF14, and MMF14_a as the PS set tests. MMF1 mainly tests the convergence and distribution ability of the algorithm when dealing with continuous and uniformly distributed Pareto sets. MMF14 mainly tests the exploration ability and robustness of the algorithm when dealing with multimodal Pareto sets, especially whether the algorithm can effectively find and maintain multiple local Pareto sets. Further test the exploration ability and robustness of MMF14_a algorithm when dealing with more complex, multimodal Pareto sets, as well as its performance in maintaining diversity and avoiding premature convergence. In the PF test, we selected MMF1, MMF12, and MMF14. MMF1 mainly tests the convergence and distribution ability of the algorithm when generating a continuous and uniformly distributed Pareto front, mainly whether the algorithm can evenly distribute solutions on the Pareto front; MMF12 mainly tests the distribution ability and adaptability of algorithms when dealing with non-uniform Pareto fronts, mainly whether the algorithm can evenly distribute solutions in regions of different densities. MMF14 mainly tests the exploration ability and robustness of the algorithm in dealing with multimodal Pareto frontiers, especially whether the algorithm can effectively find and maintain multiple local Pareto frontiers and effectively switch and explore between different modes. Different test sets test the performance of different algorithms. In the above article, the author also used different testing functions when conducting different testing items, such as CF1, CF2, CF4, and CF6 when testing parameter sensitivity and CF2, 3, 5, and 7 when generating IGD.

**Comment #4:** There is non-parametric test provided in the manuscript.

**Response #4:** Thank you for your comments. We have added Table 5 to the article. The Wilcoxon test for MODBO and other MO competitors was conducted and explained in detail.
