# Supplementary material for: Multi-objective dung beetle optimization algorithm: A novel algorithm for solving complex multi-objective optimization problems
Source: PLoS One. 2025 Oct 14;20(10):e0331713. doi: 10.1371/journal.pone.0331713 (PMC12520404; doi:10.1371/journal.pone.0331713)
Supplement: Attachment — Submitted filename: Response_to_Reviewers_auresp_5.docx [file pone.0331713.s009.docx]

**SUGGESTIONS FROM EDITOR**

**Editor:** Please review your reference list to ensure that it is complete and correct. If you have cited papers that have been retracted, please include the rationale for doing so in the manuscript text or remove these references and replace them with relevant current references. Any changes to the reference list should be mentioned in the rebuttal letter that accompanies your revised manuscript. If you need to cite a retracted article, indicate the article’s retracted status in the References list and also include a citation and full reference for the retraction notice.

**Response #1:** We have checked all the references and ensured that the format of the references meets the requirements of PLOS ONE, and we have replaced any problematic references.

**REVIEWER #3**

**Comment #1:** The introduction remains largely a list of related works without a cohesive structure or clear articulation of how this study fits into the broader context. While you mentioned adding a guiding narrative, I expected a more substantial restructuring of the section.

Further suggestion: The introduction should be reorganized, requiring changes to its structure. Focus on clearly highlighting gaps in the existing literature and explaining how your study addresses them.

**Response #1:** We have taken your valuable suggestions to heart and, as a result, have thoroughly revised the introductory section. To enhance clarity and coherence, we have restructured the content to provide a more logical flow of ideas. Additionally, we have expanded on the guiding principles to illuminate the significance and context of our research. These revisions strengthen the manuscript and better highlight the contributions of our work.

**Comment #2:** Potential Overlaps with Foundational Work

The response acknowledges similarities with foundational works but does not address the issue of textual overlap or reliance on figures from prior studies.

Further suggestion: It is critical to either rephrase sections with significant textual similarity in your own words or explicitly cite the source within the text. For any figures derived from previous works, please provide alternative visual representations or clearly attribute them to the original source.

**Response #2:** Thanks to your suggestions, we have meticulously reviewed the article and resolved all controversial graphic elements based on the feedback. We also improved the language throughout the manuscript based on a detailed test report from the checking website. These changes ensured the uniqueness of our article.

**Comment #3:** Clarity and Readability of Results and Conclusions

I do not see a significant improvement in the clarity or readability of the results and conclusions sections.

**Response #3:** We are grateful for your insightful comments, which guided us in thoroughly revising the conclusion section. This revision now provides a detailed summary of our research findings and emphasizes the significance and impact of our work. We have ensured that the conclusion effectively highlights our study's contributions and underscores its value to the field.

**Comment #4:** Research Limitations

The limitations described focus on the problem's difficulty but fail to address trade-offs or potential weaknesses in the algorithm itself. A critical self-assessment is missing, which undermines the balance of the discussion.

Further suggestion: Please provide a thorough discussion of the algorithm’s limitations, such as potential trade-offs, scalability, or computational constraints. A balanced evaluation will enhance the transparency and scientific rigor of your work.

Conclusion

**Response #4:** We appreciate your constructive comments, which have prompted us to revise the Limitations section comprehensively. In this updated section, we now provide a more detailed account of the current limitations within the Algorithms section. We have carefully considered and articulated the potential weaknesses in our algorithms, aiming to offer a transparent and honest assessment of our work.
