# Supplementary material for: Multi-objective dung beetle optimization algorithm: A novel algorithm for solving complex multi-objective optimization problems
Source: PLoS One. 2025 Oct 14;20(10):e0331713. doi: 10.1371/journal.pone.0331713 (PMC12520404; doi:10.1371/journal.pone.0331713)
Supplement: Attachment — Submitted filename: PONE-D-24-09818_R5.docx [file pone.0331713.s008.docx]

• **Introduction**:

- The introduction is now more organized and provides better context. However, it would benefit from slightly more focus on how your proposed algorithm directly addresses the gaps in the existing methods.

• **Algorithm Explanation**:

- The explanations of the Competition and Neighborhood mechanisms are clear and well-structured. No further changes are needed here.

• **Results and Discussion**:

- The results section is comprehensive and the statistical analyses are robust. However, a brief discussion on why MODBO performs slightly worse in some cases would improve balance and clarity.

• **Figures and Tables**:

- The figures and tables are clear and well-presented. Ensure that all figure captions briefly explain their relevance to the results.

• **Limitations and Future Work**:

- The updated limitations section is much improved. No additional changes are needed, but you might briefly mention specific future applications of MODBO to strengthen the conclusion.

• **Language and Style**:

- The language has improved, but a few minor typos remain (e.g., "rolling shithouse" in Section 2.1.4). Please fix these to maintain professionalism.
