# Supplementary material for: Multi-objective dung beetle optimization algorithm: A novel algorithm for solving complex multi-objective optimization problems
Source: PLoS One. 2025 Oct 14;20(10):e0331713. doi: 10.1371/journal.pone.0331713 (PMC12520404; doi:10.1371/journal.pone.0331713)
Supplement: Attachment — Submitted filename: Response_to_Reviewers_auresp_6.docx [file pone.0331713.s010.docx]

**SUGGESTIONS FROM EDITOR**

**REVIEWER #3**

**Comment #1:** Introduction: The introduction is now more organized and provides better context. However, it would benefit from slightly more focus on how your proposed algorithm directly addresses the gaps in the existing methods.

**Response #1:** Thank you for your suggestions. We have revised the content of the introduction again, adding a review of some related work and providing a detailed explanation of the necessity of our research. We also elaborated on how our work addresses existing deficiencies.

**Comment #2:** The results section is comprehensive, and the statistical analyses are robust. However, a brief discussion on why MODBO performs slightly worse in some cases would improve balance and clarity.

**Response #2:** We sincerely thank the reviewer for the constructive comments. Based on these suggestions, we have added a detailed explanation in the results analysis section to further clarify why our algorithm performed slightly worse on certain test functions, thereby enhancing the completeness and rigor of this study.

**Comment #3:** The updated limitations section is much improved. No additional changes are needed, but you might briefly mention specific future applications of MODBO to strengthen the conclusion.

**Response #3:** We appreciate your suggestions. In response, we have revised the section on future work, providing a more detailed exposition of our planned research content.

**Comment #4:** The language has improved, but a few minor typos remain (e.g., "rolling shithouse" in Section 2.1.4). Please fix these to maintain professionalism.

**Response #4:** We sincerely appreciate your valuable suggestions. In response, we have thoroughly checked all the expressions in the article and corrected any instances of spelling errors or improper phrasing.
