# Supplementary material for: Multi-objective dung beetle optimization algorithm: A novel algorithm for solving complex multi-objective optimization problems
Source: PLoS One. 2025 Oct 14;20(10):e0331713. doi: 10.1371/journal.pone.0331713 (PMC12520404; doi:10.1371/journal.pone.0331713)
Supplement: Attachment — Submitted filename: Response_to_Reviewers_auresp_7.docx [file pone.0331713.s011.docx]

**SUGGESTIONS FROM EDITOR**

**REVIEWER #3**

**Comment #1:** MODBO outperforms most algorithms but is surpassed by NSGA-II and MO_Ring_PSO_SCD in some test cases. The paper attributes MO_Ring_PSO_SCD’s success to ring topology and congestion distance but lacks a detailed explanation of MODBO’s weaknesses. A deeper analysis of these cases is needed to understand its limitations and potential improvements.

**Response #1:** Thank you for your comment. In the paper, we provide a detailed explanation of the advantages of the MO_Ring_PSO_SCD algorithm. This algorithm introduces a unique crowding distance calculation method, which effectively maintains diversity and internal uniformity when handling three-objective discontinuous multimodal functions. Although our proposed MODBO algorithm also enhances diversity and uniformity through a neighborhood mechanism, it is less flexible when dealing with these three highly deceptive functions, resulting in suboptimal performance. During the improvement process of MODBO, we considered whether to incorporate a similar mechanism. However, testing revealed that this approach excessively enhances solution distribution in the later stages of the algorithm. While such an approach does improve MODBO's performance on these three specific functions, it significantly underperforms compared to the improvement strategy proposed in this paper when applied to most test functions. Therefore, we decided not to adopt this mechanism. Our chosen improvement strategy achieves near-optimal solutions, which are acceptable for most problems. In the future, we will explore additional enhancements to the neighborhood mechanism to improve its performance on this type of test function.

**Comment #2:** MODBO introduces competition and neighborhood mechanisms, but the impact of key hyperparameters (competition intensity, archive size, neighborhood radius) is not analyzed. A sensitivity study should be included to clarify their effects on performance.

**Response #2:** We appreciate your insightful comment，In the introduction of both the competition and neighborhood mechanisms, there are no hyperparameters that require adjustment. Within the competition mechanism, selections are made from the Pareto optimal front obtained after each iteration. For each particle, it only needs to choose a guiding particle from among those closest to itself on the Pareto optimal front. Detailed explanations can be found in Section 2.2.2. Concerning the selection radius for competitive particles, this is not an issue since the Pareto optimal front changes with each iteration. The focus should be on whether there are suitable guiding particles available on this front. Regarding the neighborhood mechanism, particles are only influenced by their preceding and succeeding particles, without consideration for others, thus eliminating the concern over a neighborhood radius. The sole instance where hyperparameter settings impact algorithm performance is in the population size selection within the original DBO algorithm, which we have thoroughly tested and discussed in Fig. 2.

**Comment #3:** The explanation of MODBO’s workflow (Figure 5) is detailed but lacks pseudocode. Adding structured pseudocode would improve clarity and reproducibility.

**Response #3:** We appreciate your valuable feedback. In the final section detailing the algorithm flow, we utilized a flowchart accompanied by a detailed explanation to clearly convey the process. Introducing pseudocode in addition to these elements would have led to redundancy, as all three components—flowchart, explanation, and pseudocode—would essentially cover the same information. Recognizing this potential overlap, during the previous revisions of the paper, we decided to omit the pseudocode to streamline the presentation and avoid unnecessary repetition. We hope this approach enhances the clarity and readability of our work. Of course, we have also provided the pseudocode for your reference.

**Algorithm 1** General framework of MODBO

1. **Input: Population size** ($N_{pop}$), maximum number of iterations (${Max}_{t}$).

2. **Output:** Optimal solution.

3. Initialize algorithm parameters: Population size ($N_{pop}$), maximum number of iterations (${Max}_{t}$), population ($P_{0}$)( randomly generated)

4. Calculate the initial optimal value using ($P_{0}$)

5. **While** ($t\leq{Max}_{t}$) **do**
6. Obtain non-dominated solutions using Non-Dominated Sorting  (NDS).

7. Calculate Crowding Distance (CD).

8. Compute ($P_{j}$) Calculate Crowding Distance.

9. Combine$P_{0}$and$P_{j}:P=P_{0}\cap P_{j}$.

10. Calculate the solutions of the combined $P$.

11. Perform non-dominated sorting on $P$ based on NDR and CD.

12. Replace the original population with the generated $P$.

13. $t=t+1$

14. **End while**

**Comment #4:** The limitations section is well-written but could suggest solutions. Future work should explore hybridization with deep learning for parameter tuning, adaptation to dynamic environments for real-time optimization, and parallelization techniques for large-scale problems.

**Response #4:** We sincerely appreciate your insightful comment. We have incorporated your suggestions into the limitations section of our paper. Numerous scholars have already integrated intelligent optimization algorithms with deep learning and successfully applied these methods in various fields. In our future work, we plan to combine the MODBO algorithm with reinforcement learning to address optimization challenges in dynamic environments. Specifically, we intend to introduce an environmental monitoring mechanism and a dynamic change mechanism to effectively tackle dynamic multi-objective optimization problems.

**Comment #5:** Some sentences are awkwardly phrased or redundant. For example, "rolling shithouse" in Section 2.1.4 appears to be a typographical error and should be corrected. A thorough proofreading is needed to improve clarity and professionalism. Some figures, such as Figure 5, would benefit from clearer annotations and more descriptive captions. Figure descriptions should be revised to better explain their purpose. Some mathematical symbols and equations are used without proper definitions. All symbols and variables should be introduced before their first occurrence to ensure consistency.

**Response #5:** Thank you for your insightful comment. We have refined ambiguous and redundant expressions throughout the manuscript, ensuring clarity and precision in our writing. A thorough review of the entire paper has been conducted to eliminate any potential ambiguities and correct problematic wording. Additionally, we have revised all figure captions and table titles to enhance their clarity and consistency. All mathematical symbols used in the paper have been meticulously checked, with detailed explanations provided at their first appearance. Furthermore, we ensured that all variables in the formulas are consistent and clearly defined.
