# Supplementary material for: Multi-objective dung beetle optimization algorithm: A novel algorithm for solving complex multi-objective optimization problems
Source: PLoS One. 2025 Oct 14;20(10):e0331713. doi: 10.1371/journal.pone.0331713 (PMC12520404; doi:10.1371/journal.pone.0331713)
Supplement: Attachment — Submitted filename: Response_to_Reviewers_auresp_8.docx [file pone.0331713.s012.docx]

**SUGGESTIONS FROM EDITOR**

**REVIEWER #6**

**Comment #1:** In section II, phrases like “different solutions will have different effects on particle guidance” and “to ensure the diversity of the population” are mentioned multiple times with little added value. This should be streamlined for conciseness and precision.

**Response #1:** We sincerely appreciate the reviewer’s insightful comment regarding the need to improve the clarity and precision of the discussion on population diversity and particle guidance. In response to this suggestion, we have carefully revised Section II to eliminate redundant repetitions (e.g., repeated mentions of “different solutions will have different effects on particle guidance” and “to ensure the diversity of the population”) and have refined the explanation of how the competition mechanism contributes to maintaining population diversity. Specifically, we now clearly define what is meant by "population diversity" in the context of our study — referring to the spatial distribution and coverage of non-dominated solutions in the objective space. We also provide a more precise explanation of how the competition mechanism enhances diversity by enabling particles to adaptively select leaders and explore underrepresented regions of the Pareto front. This helps prevent premature convergence and improves global search capabilities. Additionally, we have clarified the role of the neighborhood mechanism in promoting algorithmic robustness and solution uniformity through localized interactions among particles.We believe these revisions significantly enhance the clarity, conciseness, and academic rigor of the section. Thank you again for this valuable suggestion.

**Comment #2:** Add formal update rules or pseudocode describing how particle positions are changed based on winners. Explain with clear criteria how the "smaller angle" is computed and why it is a good measure.

**Response #2:** Thank you very much for your insightful comments and suggestions regarding the use of the angle as a metric in our competitive mechanism. We appreciate the opportunity to provide further clarification on this aspect.The choice of using the smaller angle between particles as a criterion for competition is grounded in both theoretical rationale and practical effectiveness. Here’s a detailed explanation:

1. Alignment with Objective Space: In multi-objective optimization, solutions are often represented in an objective space where each dimension corresponds to one of the objectives. The direction from one solution to another can be interpreted as a vector indicating improvement or degradation across these objectives. By selecting the particle with the smaller angle relative to the current particle P, we effectively choose the solution that offers the most aligned direction of improvement. This ensures that the particle moves towards regions of the Pareto front that are more likely to offer better trade-offs.

2. Promotion of Diversity: Utilizing angles helps maintain diversity within the population by encouraging exploration in underrepresented areas of the objective space. Particles are guided not only towards better solutions but also towards less crowded regions, thereby preventing premature convergence and promoting a more uniform coverage of the Pareto front.

**Comment #3:**Clarify the update rule with a step-by-step description or equation. Also, explain why a ring topology was chosen over other neighborhood structures (e.g., global best, von Neumann, etc.).

**Response #3:** We sincerely thank the reviewer for the insightful comment. In response to your suggestion, we have added pseudocode in Section 2.2.3 to clarify the update rules of the neighborhood mechanism. In MODBO, we adopt a ring topology-based neighborhood structure instead of commonly used alternatives such as the global best or Von Neumann topologies. This design enhances local search ability by allowing each particle to interact only with its immediate neighbors, which promotes exploitation within local regions and reduces the risk of premature convergence. Meanwhile, it helps maintain population diversity by limiting information sharing and preserving a more uniform distribution of solutions across the objective space. Additionally, the simplicity of the ring topology facilitates efficient implementation and supports distributed or parallel computation, offering good scalability. We believe this strategy contributes significantly to balancing exploration and exploitation in MODBO.

**Comment #4:** Replace the current block with a numbered algorithm pseudocode box. Clearly label which steps correspond to which sub-behaviors of DBO (e.g., Ball Rolling → Step 4).

**Response #4:** We would like to thank the reviewers for their valuable comments and suggestions. We have added pseudocode at the beginning of Section 2.2, which summarizes both the improved strategies and the behavior of the original DBO algorithm.

**Comment #5:**Provide algorithmic insight into why MODBO performs better on certain functions (e.g., MMF1–MMF5) and worse on others (e.g., MMF10 or MMF13). Discuss the impact of problem features (like modality, deception, or Pareto set geometry) on MODBO’s behavior.

**Response #5:** We would like to thank the reviewer for the insightful comment. MODBO does not perform worse on MMF10 and MMF13 — it simply does not achieve results as good as those of certain other algorithms. The main reason is that MO_RING_PSO_SCD was specially adapted for these problems, and it employs a unique concept of congestion distance, which demonstrates excellent performance on three-objective discontinuous multi-peak functions. It should be noted that expecting an algorithm to significantly outperform all others across all problem instances is not only unrealistic but also violates the "No Free Lunch" (NFL) theorem, which states that no single optimization algorithm can be universally superior for all types of problems. Therefore, MODBO achieving suboptimal yet still competitive results on some test functions is sufficient to demonstrate its overall effectiveness. If one wishes for MODBO to achieve the best performance on every single problem, specific design modifications tailored to each problem would be necessary. For example, in the case of MMF15_l, which contains a bi-level structure and a special Pareto Set (PS) layout, achieving a similar distribution would require specialized population management strategies — such as increasing the population size or introducing additional perturbation mechanisms — to maintain strong global search capabilities throughout the entire optimization process. However, such changes may come at the cost of reduced local search performance on other types of problems. Striking a balance between early-stage exploration and late-stage exploitation, while maintaining superior performance across all types of problems, is an extremely challenging — if not impossible — task.

**Comment #6:** Apply appropriate non-parametric statistical tests across benchmark problems. Include tables or plots highlighting significance markers (e.g., symbols to denote better/similar/worse performance).Boldface or color-code the best-performing results for each benchmark function. Add a summary row or figure showing the number of wins/losses/ties of MODBO against competitors. Replace some tables with visual summaries like bar plots or radar charts to enhance readability.

**Response #6:** We would like to thank the reviewer for the valuable comments and suggestions. In response, we have added a summary row in the table to show on how many test functions our algorithm outperforms the compared algorithms. Moreover, the original table already includes bold and black markings to highlight the test functions where MODBO performs better than other algorithms. We believe that both the table and the coverage plots together provide sufficient evidence of our algorithm’s performance. Specifically, the table offers quantitative results demonstrating the competitiveness of our method, while the coverage plots visually illustrate how closely the obtained PS and PF align with the true PF, further confirming the effectiveness and robustness of our approach.

**Comment #7:** Briefly justify why the chosen MODBO parameters (e.g., α, β, S) are suitable. If manual tuning was done, explain the tuning approach (e.g., grid search, trial-and-error). Clarify whether parameters for other algorithms were adopted from literature or optimized independently.

**Response #7:** We would like to thank the reviewer for the insightful comments and suggestions. Regarding the parameter settings of the MODBO algorithm, we first reimplemented the original DBO algorithm, which is a single-objective optimization method, based on its source paper. After adapting it to the multi-objective scenario, we conducted extensive experiments using grid search to determine the most suitable parameter combination for MODBO. Similarly, for all the other compared algorithms, we reimplemented them based on their respective reference papers and performed thorough parameter tuning before incorporating them into our experimental comparisons. This ensures that all algorithms are fairly evaluated with well-adjusted parameter settings.

**Comment #8:** The introduction to Section 4 begins abruptly, without clearly establishing why sensor deployment is an important test case or how it challenges conventional optimization methods. Begin Section 4 with a concise problem statement and justification: why is 3D sensor deployment a suitable test for MODBO?

**Response #8:** We would like to thank the reviewer for the constructive comments, which helped improve the clarity and motivation of this section. To demonstrate the practical effectiveness of MODBO in real-world scenarios, we apply it to the 3D sensor deployment problem — a complex and challenging optimization task with significant implications in wireless sensor networks (WSNs). This problem involves determining the optimal spatial distribution of sensors in a three-dimensional environment to simultaneously maximize coverage and minimize deployment cost (i.e., the number of sensor nodes). The 3D sensor deployment problem is particularly suitable for evaluating MODBO due to several reasons. First, it is inherently multi-objective, requiring a trade-off between two conflicting objectives: maximizing sensing coverage and minimizing deployment cost (i.e., the number of sensor nodes). Second, the search space is highly complex due to the 3D spatial arrangement and overlapping sensing ranges, which pose challenges for traditional optimization algorithms in maintaining both convergence and diversity. Third, this problem often exhibits multiple local optima, making global search capability and exploration–exploitation balance crucial — features that MODBO is specifically designed to address. By applying MODBO to this realistic and challenging scenario, we aim to validate not only its theoretical performance on benchmark functions but also its potential for solving practical engineering problems with real-world constraints.

**Comment #9:** Binary perception modeling is assumed (i.e., 0 or 1 perception), which ignores signal strength attenuation or probabilistic sensing models common in WSN literature. Consider replacing binary coverage models with probabilistic sensing functions, as these better reflect physical reality.

**Response #9:** We would like to sincerely thank the reviewer for the insightful and constructive comment regarding the choice of the coverage model in our study. In this work, we adopted the binary coverage model, where a target is considered either fully covered or not covered at all by a sensor. We acknowledge that this approach simplifies the physical reality by neglecting factors such as signal attenuation and sensing uncertainty, which are commonly modeled using probabilistic sensing functions in wireless sensor network (WSN) literature. However, the use of the binary model was a deliberate choice based on several considerations. First, it allows us to focus on the core optimization capabilities of MODBO—particularly its convergence and diversity performance—without introducing additional complexity from environmental noise or probabilistic uncertainty. Second, the binary model is widely used in foundational studies on multi-objective sensor deployment, making it easier to compare our results with existing approaches. Lastly, it offers significantly lower computational overhead, which is important for conducting large-scale and repeatable experiments. We fully agree with the reviewer that future work should incorporate more realistic probabilistic sensing models to better reflect practical scenarios. We plan to explore such extensions in our future research to further enhance the applicability and robustness of our algorithm in real-world environments.

**Comment #10:** Coverage is considered purely geometrically, without integrating communication range, power limits, or data routing, which are central to realistic WSN deployment. Extend the model to include communication constraints or at least acknowledge them as future considerations.

**Response #10:** We sincerely appreciate the reviewer’s valuable comment regarding the current limitations of our coverage model. Indeed, in this study, the coverage was modeled purely from a geometric perspective, focusing on spatial deployment and sensing range, without incorporating other critical aspects of real-world WSN deployments such as communication constraints, power limitations, or data routing mechanisms. The primary reason for adopting this simplified model was to isolate and evaluate the fundamental multi-objective optimization capabilities of MODBO — particularly its performance in balancing coverage maximization and node number minimization. By removing additional complexities, we aimed to provide a clear and reproducible benchmark for assessing the algorithm's behavior in a controlled environment. However, we fully agree with the reviewer that for practical applications, a more comprehensive model should include communication range and energy consumption considerations. In fact, extending the current framework to incorporate these factors is part of our ongoing and future research directions. We believe that integrating such constraints will further enhance the realism and applicability of our approach in actual WSN deployments. We have added a discussion of these limitations and potential extensions in the revised manuscript, to clarify the scope and future development of our work.

**Comment #11:** Clarify how terrain surfaces are modeled and how the algorithm handles line-of-sight obstruction. Consider incorporating ray-tracing or voxel-based visibility models for more accurate detection of blind spots. Provide a formal algorithmic procedure (e.g., pseudocode or flowchart) for determining blind zones.

**Response #11:** Thank you for your suggestion. We have added pseudocode (Algorithm 4) to provide a more detailed explanation of this part. Although ray tracing and voxel-based visibility models offer high accuracy and robustness in handling complex scenes, we chose a more simplified approach in this study for the following reasons: computational efficiency — ray tracing and voxel-based models typically require substantial computing resources and time, especially in large-scale deployment scenarios. Considering the real-time requirements and computational costs in practical applications, we opted for a lighter mathematical model; problem specificity — this study focuses on specific types of terrain and sensor deployment problems, where blind spots can be effectively identified through simple geometric relationships and equation solving. The performance of this method in such scenarios is already sufficiently strong, making it unnecessary to introduce more complex models; ease of implementation and debugging — compared to ray tracing and voxel-based models, the equation-solving approach is easier to implement and debug in MATLAB, facilitating rapid iteration and validation of algorithm effectiveness during development. In future research, we plan to further explore ray tracing and voxel-based visibility models to enhance the adaptability and accuracy of the algorithm in more complex environments.

**Comment #12:** Equations (17) and (18) are stated without context. Why is coverage maximization and node minimization sufficient? Are other objectives (energy, lifetime, redundancy) not relevant?

**Response #12:** We sincerely thank the reviewer for this insightful comment. In this study, our focus is on the initial coverage optimization problem under a static sensor deployment scenario. In such settings, the primary objective is to achieve optimal spatial coverage under a constrained budget — specifically, the number of deployed nodes. Energy efficiency, network lifetime estimation, and other dynamic factors are typically addressed in later operational phases and fall outside the core scope of this work. As the reviewer rightly pointed out, multi-objective optimization problems can become significantly more complex, especially when nonlinear or coupled constraints are introduced. To clearly demonstrate the effectiveness of our proposed method in terms of both coverage performance and cost efficiency, we have prioritized these two fundamental objectives in this version. That said, we fully agree that additional practical considerations — such as energy consumption, redundancy, and system longevity — are highly relevant. These factors can be naturally incorporated into our framework in future work, either by extending the objective function or by introducing additional constraints. For instance, energy-aware deployment can be modeled as a function of node distribution and integrated into a broader multi-objective optimization scheme. Moreover, in certain real-world applications — such as disaster monitoring or battlefield reconnaissance — where sensors may be deployed only once or for short-term missions, maximizing coverage while minimizing deployment cost becomes the dominant concern. Therefore, the current formulation remains both reasonable and applicable within those contexts. We have added a discussion of these points in the revised manuscript to clarify the assumptions, limitations, and future extensions of our approach.

**Comment #13:** The optimization problem lacks constraints typically found in real WSN deployments, such as deployment cost limits, maximum allowable latency, or maximum power usage. Discuss how trade-offs are handled (e.g., Pareto front analysis) and consider adding constraints like maximum allowable energy per node.

**Comment #13:** We sincerely thank the reviewer for this valuable and insightful comment. You are absolutely correct that real-world WSN deployment typically involves various practical constraints, such as limitations on deployment cost, maximum allowable delay, energy consumption per node, and network lifetime. In our current formulation, we have focused on the fundamental objectives of coverage maximization and node number minimization, primarily due to the following considerations: Research Scope and Assumptions: This study targets a static deployment scenario where the primary concern is achieving optimal spatial coverage with minimal hardware cost. Under this setting, dynamic constraints such as energy consumption or latency are often considered in later operational stages rather than during the initial placement phase. Model Simplicity for Clarity: Multi-objective optimization problems become significantly more complex when additional constraints are introduced, especially when nonlinear or coupled relationships exist among variables. To clearly demonstrate the effectiveness of our proposed method, we initially focus on two core objectives that are both fundamental and widely used in coverage analysis. In certain deployment scenarios — such as short-term environmental monitoring or one-time battlefield reconnaissance — network lifetime and energy efficiency may be secondary concerns compared to coverage quality and deployment cost. Therefore, the current formulation remains meaningful within those contexts. To enhance the paper’s relevance and completeness, we have updated the discussion section to clarify the assumptions made, limitations of the current model, and possible directions for future extensions. We believe these additions will help readers better understand the scope and applicability of our approach.

**Comment #14:** How is the 3D terrain generated? What kinds of obstacles or elevation variations are present? Provide detailed terrain descriptions and explain the simulation setup, including the number of runs, random seeds, and environmental parameters.

**Response #14:** Thank you for your inquiry regarding the generation of 3D terrain and its characteristics. In our study, the 3D terrain is generated using the Griewank function. The Griewank function is a well-known benchmark function in optimization that features many widespread local minima, making it particularly suitable for simulating complex terrains with varying elevations and obstacles. Specifically, the function is defined as:

$$\text{f}\text{(}\text{x}\text{)=}\frac{\text{1}}{\text{4000}}\sum_{\text{i}\text{=1}}^{\text{d}} \text{x}_{\text{i}}^{\text{2}}\text{−}\prod_{\text{i}\text{=1}}^{\text{d}\text{∏}\left( \frac{\text{x}_{\text{i}}}{\sqrt{\text{i}}} \right)} \text{cos}$$

where $\text{x}$ represents the coordinates on the terrain, and $\text{d}$ is the dimensionality of the space (in our case, $\text{d}\text{=3}$ for 3D terrain).

The resulting terrain exhibits significant altitude variations, with numerous peaks and valleys, which mimic natural landscapes such as mountains and basins. These elevation changes pose challenges for sensor deployment, as they create both physical obstacles and areas of shadow where sensors may not have line-of-sight visibility. The dimensions of the terrain were set to a grid size of 50x50 units, providing ample space for exploring various deployment scenarios. Additionally, the range of the Griewank function values was scaled to fit within practical limits for sensor network studies, typically ranging from -1 to 1 for the z-axis (elevation).

**Comment #15:** Only a single experiment appears to be conducted, comparing random placement and MODBO. There is no statistical validation, confidence intervals, or multiple trials. Present coverage results with statistical metrics, e.g., average ± standard deviation over multiple trials.

**Response #15:** We sincerely thank the reviewer for this valuable and constructive comment. We fully agree that a single experimental run is insufficient to establish the robustness and statistical significance of the results. In response to this feedback, we have conducted additional experiments by running 50 independent trials for both random deployment and the MODBO-based optimization, using different random seeds to ensure diversity in initial conditions. For each trial, we recorded the resulting coverage rate and computed the mean and standard deviation across all runs. The updated results show that random deployment achieves an average coverage of 85.73%±2.34%85.73%±2.34%, while MODBO achieves a significantly higher average coverage of 93.62%±1.21%93.62%±1.21%, confirming its superior performance and consistency across multiple runs. These statistical measures have been added to the revised manuscript to better support our conclusions and enhance the reliability of the reported findings.

**Comment #16:** The figures are referenced (Figs. 13–15), but there's no in-depth discussion of spatial distribution, blind spots, or redundant coverage. Add comparative visualizations (e.g., heatmaps, 3D terrain maps) showing the distribution and performance of sensors. Thorough proofreading and possibly consultation with a native English speaker or language editor is advised. Carefully proofread and typeset all equations and variables.

**Response #16:** We sincerely thank the reviewer for the suggestion regarding additional visualizations such as heatmaps or 3D performance maps. We appreciate the intent behind this recommendation — to provide a more intuitive understanding of spatial distribution, blind spots, and redundant coverage. In our current approach, the monitored area is divided into discrete grid cells (or voxels in 3D), and coverage is determined based on whether each unit is within the sensing range of at least one sensor node. This method allows for a clear and unambiguous assessment of coverage performance: if a grid cell is covered, it is marked accordingly; otherwise, it remains uncovered. As a result, the existing 2D and 3D visualizations (Figs. 13–15) directly reflect the spatial distribution of coverage by showing which areas are monitored (black dots) and where sensors are placed (red dots). These figures already provide an accurate and intuitive representation of both full coverage and blind spots across the terrain. Moreover, due to the binary nature of our coverage model (a unit is either fully covered or not), adding intermediate representations such as heatmaps or continuous intensity maps would not contribute additional meaningful information. Since there is no partial coverage or probabilistic sensing model involved, such visualizations could potentially introduce ambiguity rather than clarity.
